# Uncertainty-aware posture quality assessment with Bayesian liquid neural networks

**DOI:** 10.3389/fnbot.2026.1958419

**Published:** 2026-10-02

**Authors:** Yuhua Mu, Mingliang Zhu, Zhi Tao

**Affiliations:** 1 Shaoxing Institute of Technology, Zhejiang, China; 2 College of Big Data, Chongqing Polytechnic Institute, Chongqing, China; 3 Factory Vocational and Technical School, Guangzhou, Guangdong, China

**Keywords:** Bayesian inference, liquid neural networks, posture quality assessment, reliable prediction, uncertainty quantification

## Abstract

Automated posture quality assessment has become increasingly important in health and wellness technology. However, existing methods predominantly provide point estimates without quantifying prediction confidence, posing a critical limitation in safety-sensitive applications where incorrect assessments may lead to improper guidance. This paper addresses this gap by introducing a reliability-oriented evaluation framework based on Bayesian Liquid Neural Networks (LNNBI). Unlike conventional approaches, our method simultaneously delivers a quality score and a principled uncertainty measure derived from Bayesian variational inference. More importantly, we incorporate a rejection mechanism that enables the model to abstain from making predictions when uncertainty exceeds a predefined threshold, establishing a built-in safeguard for practical deployment. To support this framework, we construct a longitudinal dataset comprising 50 participants over 200 sessions, decomposing four fundamental human postures into seven biomechanical indicators. Experimental results demonstrate that LNNBI achieves 0.8715 accuracy and 0.9105 precision, outperforming LSTM, MC-GRU, TSGCN, and YoNet baselines. Uncertainty analysis reveals a strong correlation between prediction error and uncertainty (Pearson’s *r* = 0.73), confirming that the model reliably identifies its own low-confidence predictions. With a 15% rejection rate, the effective accuracy reaches 0.923. Correlation analysis further identifies Pelvic Position and Neutrality (*r* = 0.682) and Spinal Alignment (*r* = 0.595) as the most influential biomechanical indicators. This work offers a reliability-first perspective on automated posture assessment, with promising potential for deployment in personalized health coaching and remote guidance, particularly on GPU-accelerated systems.

## 1 Introduction

In recent years, the global surge in health consciousness has significantly propelled the adoption of posture-based wellness practices for holistic health maintenance. Such practices enhance physical capabilities—including flexibility, strength, and balance—while simultaneously fostering mental clarity and stress reduction. However, the therapeutic efficacy and safety of these activities are fundamentally contingent upon the precision of biomechanical execution. Incorrect alignment can not only negate intended benefits but also precipitate musculoskeletal strain and injury. Consequently, ensuring correct form remains paramount, traditionally requiring the vigilant supervision of experienced instructors. This necessity presents a significant challenge for individuals practicing independently, beyond the structured environment of supervised settings.

The convergence of technological innovation and the pervasive demand for guided practice has stimulated substantial research into computational solutions. Deep learning has emerged as a powerful paradigm for automating human posture estimation, making it ideally suited for movement quality analysis. Foundational studies established Convolutional Neural Networks (CNNs) as dominant architectures for pose recognition, subsequently inspiring specialized model developments. Amira (Pereira et al., 2026) introduced a deep learning framework for posture estimation through biomechanical attribute analysis. Meng and Shi (2025) developed a neural network optimized for multi-class posture classification. Xie and Wang (2025) implemented a hybrid CNN-MediaPipe architecture for improved skeletal keypoint extraction. Huong et al. (2026) conceptualized a real-time system delivering corrective feedback. More recently, researchers have explored Liquid Neural Networks and Bayesian Deep Learning for dynamic motion analysis and uncertainty quantification in human movement estimation tasks.

Despite these advances, a significant limitation pervades existing posture quality assessment systems: they produce point estimates without quantifying prediction confidence. In safety-sensitive applications such as physical training and rehabilitation, an incorrect assessment is not merely inaccurate—it can lead to improper corrections, increased injury risk, and erosion of user trust. A model that confidently delivers a wrong score is arguably more problematic than one that acknowledges its uncertainty. The consequences are particularly acute in unsupervised practice scenarios, where users lack expert verification and may unknowingly adopt incorrect corrections.

The consequences of overconfident incorrect feedback are not merely theoretical. Clinical case studies in sports rehabilitation have documented that unsupervised exercise following generic instructional content can induce substantial musculoskeletal injury. A 45-year-old male with no prior spinal pathology developed severe lumbopelvic pain (Visual Analogue Scale 8.5/10) requiring 45 days of supervised physiotherapy after regular unsupervised stretching with improper technique; the condition was attributed to abnormal biomechanical stress, neural irritation, and soft tissue microtrauma resulting from faulty movement execution (Kaushik and Singh, 2026). Epidemiological evidence from a large-scale international survey of 3,668 users of a bodyweight training app—where all instruction is delivered without professional supervision—revealed that 24% of participants sustained at least one injury over a 9-month period, with an injury incidence rate of 4.57 per 1,000 training hours (Hertel et al., 2022). Muscle strains (28.5%), muscle injuries (14.4%), and insertional tendinopathies (12.1%) were the most frequently reported conditions. Critically, 35% of injured respondents believed their injury could have been prevented by better technique, underscoring the direct link between unsupervised, incorrectly executed movements and adverse outcomes (Hertel et al., 2022). Beyond acute injury, incorrect automated feedback in unsupervised settings may reinforce maladaptive movement patterns that increase long-term joint loading, as biomechanical analyses have shown that altered knee kinematics—such as excessive valgus collapse during squatting—can increase medial compartment contact forces by over 50% compared to correct sagittal-plane alignment (Samaan et al., 2019). From a pedagogical perspective, poorly calibrated feedback—especially when delivered with unwarranted confidence—may narrow learners’ attentional focus, encourage “score-chasing” behavior over genuine movement quality, and foster dependence on external cues at the expense of intrinsic movement awareness (Johari and Mukherjee, 2026). These documented harms underscore a critical imperative: for automated posture assessment deployed in unsupervised practice scenarios, a model that confidently delivers a wrong score is not merely inaccurate—it is actively hazardous. This recognition motivates the need for systems that can explicitly quantify their own prediction confidence and abstain when that confidence is insufficient. Therefore, we argue that reliability is as important as accuracy for automated posture assessment systems, and that predictive uncertainty should be explicitly modeled and reported.

To address this gap, we propose an uncertainty-aware posture quality assessment framework based on Bayesian Liquid Neural Networks (LNNBI). Liquid Neural Networks (LNNs) offer distinctive advantages for modeling skeletal time-series data, which are central to posture quality assessment. Unlike conventional RNNs (e.g., LSTM or GRU) that operate with fixed discrete time steps, LNNs are governed by continuous-time dynamics with input-dependent time constants. This property enables two critical capabilities. First, the network automatically adjusts its temporal resolution in response to input variations—accelerating its response during rapid transitions and maintaining stability during slow, sustained postural holds—without requiring heuristic down sampling or interpolation. Second, unlike Neural ODEs that apply globally shared dynamics across all inputs, LNNs modulate their state evolution based on incoming sensory signals, providing context-aware temporal representation. These characteristics make LNNs particularly suitable for human motion analysis, where movements exhibit highly variable speeds, irregular rhythms, and multi-scale temporal structures that challenge both discrete-step RNNs and input-independent continuous-time models. Moreover, Bayesian variational inference allows us to quantify predictive uncertainty in a principled manner, transforming the model from a “black-box scorer” into a “reliable decision assistant.” More importantly, we introduce a rejection mechanism: when predictive uncertainty exceeds a predefined threshold, the model abstains from making a prediction and signals the need for human verification. This built-in safeguard prevents potentially harmful incorrect predictions from reaching the user and identifies samples that would benefit from expert review.

To empirically validate our approach, we construct a longitudinal dataset of 50 practitioners over 200 consecutive days. We decompose four fundamental postures representing distinct biomechanical demands—spinal extension, spinal flexion with elongation, unilateral loading, and single-limb balance—into seven biomechanical performance indicators, each quantitatively scored based on expert assessment. The resulting dataset captures both cross-sectional movement quality and temporal evolution of individual performance, providing a rich foundation for training and evaluating temporal models.

Contributions: the main contributions of this work are:

1. We propose an uncertainty-aware posture quality assessment framework that provides both quality scores and confidence measures, establishing a new dimension of reliability beyond conventional accuracy-oriented approaches.
2. We introduce a rejection mechanism that enables the model to abstain from low-confidence predictions, establishing a built-in safeguard for practical deployment and enabling human-in-the-loop decision making.
3. We construct a longitudinal dataset of 50 participants across 200 days, capturing the temporal dynamics of movement performance through seven biomechanical indicators.
4. Through systematic correlation analysis, we identify and rank the biomechanical indicators most influential to movement quality, providing actionable insights for practitioners and instructors.

The remainder of this paper is organized as follows. Section 2 reviews related work. Section 3 presents the proposed methodology, including the biomechanical indicators, dataset construction, and the LNNBI framework with rejection mechanism. Section 4 describes experiments and results. Section 5 discusses the findings. Section 6 concludes the paper.

## 2 Related work

Automated posture quality assessment occupies an expanding intersection of computer vision, machine learning, and biomechanics. Recent methodological advances have explored diverse approaches ranging from traditional feature engineering to sophisticated deep learning paradigms and multimodal sensing integration. This section reviews current state-of-the-art, emphasizing vision-based techniques, advanced neural architectures, and Bayesian uncertainty quantification methods.

### 2.1 Evolution of vision-based human posture estimation

Early human posture estimation research predominantly relied on conventional computer vision techniques with hand-crafted feature representations. A prominent approach utilized angle-based features extracted from skeletal key points. Ranjith et al. (2026) employed computer vision to extract angular features from human postures, subsequently classifying them using various machine learning algorithms, with Extremely Randomized Trees demonstrating superior predictive accuracy. This approach highlights geometric features’ viability for posture classification, though robustness limitations may emerge for complex, dynamic sequences.

The field subsequently evolved through adoption of deep learning-based pose estimators including OpenPose and MediaPipe. Ke et al. (2024a) employed MoveNet preprocessing for keypoint extraction from posture images, followed by Artificial Neural Network classification. Similarly, another investigation leveraged OpenPose for 136-point skeletal feature extraction to train Random Forest classifiers, emphasizing the importance of evaluating models on unseen subjects and varying camera angles to ensure generalizability (Joshita Reddy et al., 2025). Integration of real-time pose estimation frameworks has substantially streamlined feature extraction pipelines.

Further advancing real-time capabilities, Mishra (Thombare and Gumaste, 2025) implemented a computer-assisted movement instruction system using TOF camera and Kinect V2.0 for real-time data acquisition. The study employed motion redirection algorithms and keyframe interpolation for optimized action recognition, demonstrating that joint angle values effectively identified substandard coaching movements.

Enhancing practical real-time systems, (Lu et al., 2025) proposed a movement quality evaluation system for enhanced educational applications. Complementing this, a hybrid deep learning model combining CNNs and LSTMs was developed for video-based action recognition (Li et al., 2024). The CNN extracted spatial features from OpenPose keypoints while the LSTM captured temporal dependencies, achieving 97.38% testing accuracy, underscoring spatiotemporal models’ advantages for motion sequence analysis.

### 2.2 Liquid neural networks and Bayesian deep learning for dynamic analysis

Modeling temporal dynamics of human movement necessitates specialized neural architectures. Liquid Neural Networks have emerged as powerful tools for time-series processing due to their continuous-time dynamics and adaptive input response characteristics. Joshita Reddy et al. (2025) explored LNNs for time-series pose forecasting, demonstrating capability in capturing human movement dynamics essential for analyzing movement flows and transitions. While Bayesian RNNs have demonstrated effectiveness in motion analysis with uncertainty quantification, their reliance on fixed temporal discretization limits their ability to handle variable-speed movements—a common challenge in real-world practice scenarios where practitioners exhibit different tempos, skill levels, and fatigue-induced speed variations. Neural ODEs, though offering continuous-time modeling, employ input-independent dynamics that cannot adapt to the changing characteristics of motion signals. In contrast, LNNs bridge this gap by combining continuous-time representation with input-adaptive dynamics: the time constant τ in the liquid state equation is modulated by the input itself, allowing the network to naturally accommodate irregularly sampled or variable-speed sequences. This property is particularly advantageous for posture quality assessment, where movements span from rapid transitions to sustained static holds, and where individual differences in execution speed are informative of skill proficiency. The Bayesian extension of LNNs further enhances this capability by providing principled uncertainty estimates over both network parameters and temporal predictions, offering a complete framework for reliable posture evaluation. Building on this, Lee et al. (2025) proposed Liquid Time-Constant Networks for adaptive motion modeling, further enhancing capacity to handle variable-speed movements typical of personalized physical training.

A significant limitation of deterministic deep learning models is their inability to quantify predictive uncertainty. Bayesian Deep Learning addresses this by treating model parameters as probability distributions. Jain et al. (2024) presented a Bayesian Deep Learning framework for human pose estimation under uncertainty, critical for identifying low-confidence predictions in suboptimal conditions such as occlusion or poor illumination. This approach extends to recurrent architectures through Schmidt et al. (2024), who integrated Bayesian inference into Recurrent Neural Networks for improved uncertainty quantification in motion analysis. For complex graph-based representations, Liu et al. (2024) developed uncertainty-aware pose estimation using Bayesian Graph Neural Networks, effectively propagating uncertainty through human skeleton graph structures. Xu et al. (2024) applied Bayesian Variational Inference to Dynamic Neural Networks for human activity recognition, enhancing model robustness—a highly desirable property for real-world movement assessment systems.

### 2.3 Skeleton-based action recognition and multimodal applications

Skeleton-based action recognition has benefited from modeling key movement events rather than isolated frames. Li et al. (2024) developed methods for complex action classification through intrinsic event group modeling. A comprehensive survey by Gao et al. (2025) detailed applications of event-based approaches in sports training, underscoring their potential for structuring sequential movement analysis.

No single sensing modality is universally optimal. Vision systems may suffer from occlusion, while wearable sensors provide direct kinematic data but require hardware. Multimodal fusion consequently has gained traction. Shen et al. (2026) proposed a movement analysis system fusing wearable sensor and camera data, leveraging complementary strengths for robust assessment. On the hardware front, Rahman et al. (2024) designed a smart mat with embedded sensors providing haptic and visual posture feedback, representing a tangible Internet of Things application in personalized physical training.

Beyond pose identification, growing research addresses in-depth biomechanical analysis. Abbas et al. (2025) conducted deep learning-based biomechanical analysis of human postures, quantifying key postural indicators and their musculoskeletal impact. Lin et al. (2025) performed 3D kinematic evaluation assessing movement practice effects on segmental trunk control, providing scientific basis for therapeutic benefits. Gaikwad et al. (2025) constructed neural network models for exercise load monitoring based on movement training data, demonstrating applications in personalized training and rehabilitation. Systematic reviews, such as Rajarajan et al. (2025), consolidate knowledge in this rapidly evolving field by summarizing health benefits, research trends, and technological intervention efficacy for movement-based wellness practices.

## 3 Methodology

This section presents a comprehensive methodology for posture quality assessment. First, we define seven key performance indicators for evaluating fundamental human postures. Subsequently, we describe the longitudinal dataset construction and partitioning. Finally, we introduce our model, the Liquid Neural Network with Bayesian Inference (LNNBI), which synergistically combines LNN’s dynamic processing strengths with Bayesian inference’s principled uncertainty quantification and rejection mechanism.

### 3.1 Biomechanical performance indicators

Based on biomechanical principles fundamental to human movement, we identify and define seven distinct indicators for comprehensive evaluation of four fundamental postures representing distinct biomechanical demands: spinal extension, spinal flexion with elongation, unilateral loading, and single-limb balance. These metrics serve as critical criteria for evaluating execution accuracy, efficacy, and safety. A detailed breakdown is systematically presented in Table 1.

**Table 1:** Summary of posture quality performance indicators.

indicator	Assessment focus	Application examples in poses
SAE	Maintaining the spine’s natural curvature, avoiding lumbar strain.	Spine straight, elongated towards hips. Backbend initiated by the thoracic spine.
SGSP	Scapula position and stability, shoulders down, avoiding elevation.	Supports upper body weight. Enables strong, sustained arm extension.
CME	Activation of abdominal and deep core muscles to stabilize the torso.	Essential for maintaining balance. Stabilizes the pelvis and upper body.
PPN	Pelvis in a neutral position, avoiding excessive anterior or posterior tilting.	Avoids anterior tilt lumbar compression. Pelvis remains level and broad.
LBJAS	Correct alignment of knees, ankles, and hips.	Front knee at 90° angle, aligned with second toe. Standing leg’s knee is soft, not locked.
FFWD	Even weight distribution and stable contact with the ground.	Weight even across three points of the foot. Heels press down as hands push in.
BRC	Smooth, deep breaths coordinated with movement and holding.	Inhalation assists spine elongation and lift. Deep, steady breaths maintain endurance and focus.

*Indicator 1*. Spinal Alignment and Elongation (SAE). This indicator evaluates whether the spine maintains its natural physiological curvature or is effectively elongated, avoiding excessive arching (hyperextension) or rounding of the lumbar spine and creating uniform extension or back bending where required.

*Indicator 2*. Shoulder Girdle Stability and Positioning (SGSP). This assesses the stability and positioning of the scapulae. The shoulders should be drawn away from the ears (avoiding elevation), with the scapulae slightly retracted and drawn down the back to create space and strength in the joints.

*Indicator 3*. Core Muscle Engagement (CME). This indicator measures the activation of the abdominal and deep core muscles. Engaging the core stabilizes the torso, protects the lumbar spine, and integrates the strength of the upper and lower body.

*Indicator 4*. Pelvic Position and Neutrality (PPN). This evaluates whether the pelvis is in a neutral position, avoiding excessive anterior or posterior tilting. As the body’s power center, the pelvic position directly influences the alignment of the spine and legs.

*Indicator 5*. Lower-Body Joint Alignment and Stability (LBJAS). This focuses on the alignment relationship between the knees, ankles, and hips. The knees should track in the same direction as the toes and should not extend significantly beyond the ankles to prevent joint strain.

*Indicator 6*. Foot Foundation and Weight Distribution (FFWD). This assesses the contact area of the feet with the ground and the even distribution of body weight. A stable “foundation” is fundamental for all standing and balancing postures, ensuring the effective upward transmission of force.

*Indicator 7*. Breathing Rhythm and Coordination (BRC). This indicator evaluates whether the breath is smooth, deep, and coordinated with movement. Breath regulation is essential in many physical practices; it calms the nervous system, increases endurance, and facilitates deeper engagement.

### 3.2 The dataset

To empirically validate the proposed methodology, a dedicated and structured dataset was meticulously constructed. The data collection process, composition, and preparatory steps are detailed as follows. A tabular view of a subset of this data is presented in Table 2, illustrating the score for each indicator for every participant across different days.

**Table 2:** Detailed structure of the posture quality performance dataset.

Day	Indicators	*S_1_* (score)	…	*S_i_* (score)	…	*S_50_* (score)
1	SAE	63.7	…	score	…	50.54
1	SGSP	74.11	…	score	…	53.04
1	CME	57.74	…	score	…	50
1	PPN	57.14	…	score	…	50
1	LBJAS	60.23	…	score	…	50
1	FFWD	60.82	…	score	…	50
1	BRC	68.13	…	score	…	50
2	SAE	59.61	…	score	…	57.68
…	…	…	…	score	…	…
200	BRC	55.81	…	score	…	69.78

Item *S_i_* indicates that *i*-th participant obtains the score in the *j*-th day.

#### 3.2.1 Participant demographics

The 50 participants in this study were university students recruited from a physical education course, comprising 32 females (64%) and 18 males (36%), aged 18 to 26 years (mean ± SD: 20.8 ± 2.1 years). Baseline anthropometric measurements yielded a mean BMI of 21.9 ± 2.3 kg/m^2^ (range: 18.4–26.5 kg/m^2^), with 43 participants (86%) classified as normal weight (18.5–24.9 kg/m^2^) and 7 participants (14%) as overweight (25.0–26.5 kg/m^2^); no participants were underweight or obese. Self-reported physical activity levels, assessed using the International Physical Activity Questionnaire (IPAQ) short form, indicated that 28 participants (56%) engaged in moderate physical activity (e.g., recreational sports 1–3 times per week), 17 participants (34%) in high physical activity (e.g., regular sports training ≥4 times per week), and 5 participants (10%) in minimal physical activity (less than one structured exercise session per week). All participants reported no history of musculoskeletal disorders, spinal pathologies, or acute injuries that would contraindicate participation in the postural exercises examined in this study, and all provided written informed consent prior to data collection.

#### 3.2.2 Performance indicators and scoring

Each participant’s execution of four fundamental postures was evaluated daily against seven predefined indicators. For each posture, indicators were independently scored based on video recordings from three camera angles (frontal, sagittal, dorsal) using standardized placements. Three certified instructors (5–10 years teaching experience) independently reviewed recordings and assigned scores using a standardized rubric. Final scores were computed as the average of three ratings. Inter-rater reliability was assessed using the intraclass correlation coefficient [ICC(2,1)], yielding values from 0.82 to 0.91 across indicators, indicating good to excellent agreement. The daily indicator vector represents the average of the four postures’ indicator scores, providing a holistic daily performance profile for each participant.

#### 3.2.3 Data structure and representation

The resulting dataset is organized as a three-dimensional matrix, encapsulating the essence of the longitudinal study. Its structure can be conceptualized as follows,

- *Dimension 1*: Time. This axis represents the 200 consecutive days of data collection. Each day corresponds to a complete set of video recordings for all four poses from each participant.
- *Dimension 2*: Participants. This axis corresponds to the 50 unique participants (S1 to S50).
- *Dimension 3*: Features. This axis comprises the 7 performance indicator scores for each participant on each day, averaged across the four poses.

#### 3.2.4 Dataset partitioning

To rigorously evaluate the model’s performance, the complete dataset was partitioned into two mutually exclusive sets.

- *Training set (80%)*: This subset, containing data from 40 randomly selected students and the initial 160 days for the remaining 10, was used exclusively to train the LNNBI model and optimize its parameters.
- *Testing set (20%)*: This held-out subset, comprising data from the other 10 students and the final 40 days for the training participants, was used solely for the final evaluation. It tests the model’s ability to generalize to new students and to forecast future performance, thus verifying its practical utility and robustness.
- *Normalization*: All indicator scores were normalized to a [0, 1] scale using Min-Max scaling applied across the entire dataset (i.e., using the global minimum and maximum values for each indicator computed from the training set). This ensures that all features contribute equally to the model training process, preventing any single indicator from dominating due to its original scale, while also preventing information leakage from the test set.

To further disentangle the two generalization dimensions—cross-subject generalization and temporal forecasting—we designed two additional evaluation protocols:

- *Cross-subject protocol*: Training on 40 participants (all 200 days) and testing on the remaining 10 unseen participants (all 200 days). This evaluates the model’s ability to generalize to new individuals.
- *Temporal protocol*: Training on all 50 participants (first 160 days) and testing on the same 50 participants (last 40 days). This evaluates the model’s ability to forecast future performance for known individuals.

These protocols were applied in addition to the main mixed-split evaluation, allowing us to separately quantify the model’s strengths in each dimension.

### 3.3 System operational workflow

#### 3.3.1 Workflow

To provide clear understanding of how the proposed framework operates in practice, we describe the end-to-end workflow from raw data acquisition to final posture quality assessment:

*Stage 1*: Data Acquisition. For each training session, participants perform four fundamental postures while recorded from three synchronized camera angles at 30 fps with 1920 × 1,080 resolution.

*Stage 2*: Pose Extraction and Keypoint Detection. Recorded videos are processed using MediaPipe or OpenPose to extract 33 skeletal keypoints per frame, used to compute joint angles, segment orientations, and body part positions relative to anatomical reference planes.

*Stage 3*: KPI Quantification. For each posture and indicator, algorithmic formulas translate skeletal keypoints into quantitative scores:

- *SAE*: Computed as deviation of spine curvature from ideal neutral curve, measured using angles between thoracic, lumbar, and sacral segments.
- *PPN*: Calculated as pelvic tilt angle relative to horizontal plane, scored inversely to absolute deviation from neutral (0°-10° range).
- *CME*: Inferred from trunk kinematic stability and abdominal-to-lumbar muscle activation patterns derived from movement smoothness. When algorithmic estimation is ambiguous (e.g., occlusion), scores are flagged for manual verification by certified instructors.

*Stage 4*: Event Group Formation. Continuous movement sequences are segmented into meaningful movement phases. For each posture, key transition events (entry, peak hold, and exit) are identified, with indicator scores aggregated to produce representative vectors.

*Stage 5*: model inference. Aggregated indicator vectors are fed into trained LNNBI model, outputting predicted overall performance score (0–100 scale) and predictive uncertainty (posterior predictive standard deviation).

*Stage 6*: result interpretation and feedback. Output score and uncertainty estimation are displayed to practitioners or instructors. High uncertainty predictions trigger recommendations for manual reassessment, while low-confidence scores automatically flag specific indicators requiring correction.A note on vision-based physiological indicators. Among the seven KPIs, BRC (breathing rhythm) and CME (core muscle engagement) are inherently internal physiological states that cannot be directly measured from RGB video. Their extraction from skeletal key points relies on indirect proxies—respiratory rate from chest expansion periodicity and core activation from trunk kinematic stability. While these proxies are not equivalent to ground-truth measurements (e.g., electromyography for CME or respiratory inductance plethysmography for BRC), we include them for three reasons: (1) they are widely used as practical substitutes in video-based human movement analysis when direct physiological sensors are unavailable; (2) their inclusion allows the model to leverage any informative signal that can be extracted from the available data, even if imperfect; and (3) their lower but non-negligible correlation (*r* = 0.5078 for BRC and *r* = 0.5847 for CME) confirms that they do contribute some predictive information. Nevertheless, we explicitly acknowledge in Section 5.4 that vision-only estimation of these internal states has inherent limitations, and multimodal sensor integration is a key direction for future work.

#### 3.3.2 Mathematical formulation of biomechanical indicators

Following keypoint extraction, each biomechanical indicator was quantified using mathematical formulas derived from skeletal geometry. For three keypoints *A*, *B*, and *C*, the joint angle at *B* was computed via using the cosine law:

$$\theta =\cos^{-1}(\frac{\vec{\mathrm{AB}}\cdot \vec{\mathrm{BC}}}{∥\vec{\mathrm{AB}}∥∥\vec{\mathrm{BC}}∥})$$

where $\vec{\mathrm{AB}}=(x_{B},-x_{A}y_{B}-y_{A})$ and

$\vec{\mathrm{BC}}=(x_{C},-x_{B}y_{C}-y_{B})$ . Here, the limb lengths were computed as Euclidean distances.

$$d_{\mathrm{ij}}=\sqrt{{\left(x_{j}-x_{i}\right)}^{2}+{\left(y_{j}-y_{i}\right)}^{2}}$$

Each KPI was then defined as:

- *SAE*: Evaluated as the deviation of the spinal curvature from an ideal neutral curve, defined as the average of thoracic, lumbar, and sacral joint angles relative to reference positions.
- *SGSP*: Defined as the vertical displacement of the shoulder landmarks (11, 12) from the neck landmark (0), normalized to the shoulder-to-neck distance.
- *CME*: Inferred from trunk kinematic stability over a temporal window, defined as the inverse of the variance of pelvic angle and trunk inclination across frames.
- *PPN*: Calculated as the tilt angle between the line connecting left and right hip landmarks (23, 24) and the horizontal axis, scored inversely to its absolute deviation from neutral-10.
- *LBJAS*: Defined as the angular deviation of the knee joint (landmarks 25, 26) from the ideal sagittal-plane alignment, computed from hip-knee-ankle angles.
- *FFWD*: Assessed as the symmetry of foot landmark (27, 28) distances from the pelvic center projection.
- *BRC*: Estimated from the periodic expansion and contraction of the torso bounding box over time.

All indicator scores were normalized to a [0,100] scale. For indicators requiring temporal stability (CME, BRC), a sliding window of 30 frames was applied with a stride of 10 frames.

#### 3.3.3 Signal processing for temporal indicators (BRC and CME)

Unlike static indicators (e.g., PPN, SAE) that are computed from single-frame keypoint geometry, BRC (Breathing Rhythm and Coordination) and CME (Core Muscle Engagement) are temporal indicators that rely on keypoint dynamics across frames. Their estimation from RGB video at 30 fps is susceptible to motion artifacts from body sway, limb movement, and keypoint detection jitter. To address this, we implemented a multi-stage signal processing pipeline:

For BRC estimation, the chest center landmark (MediaPipe landmark 0, or the centroid of landmarks 11–12) was tracked over a sliding window of 150 frames (5 s). The vertical displacement signal was filtered using a zero-phase low-pass Butterworth filter (cutoff frequency: 0.5 Hz) to remove high-frequency jitter, followed by a high-pass filter (cutoff: 0.1 Hz) to remove baseline drift due to postural shifts. The resulting signal was subjected to peak detection with a minimum inter-peak interval of 1.5 s (corresponding to a maximum respiratory rate of 40 breaths/min) to exclude false peaks from non-respiratory motion artifacts. Respiratory rate was computed as the number of valid peaks per minute, and rhythmicity was quantified as the coefficient of variation of inter-peak intervals.

For CME estimation, trunk stability was assessed via the torso inclination angle (defined as the angle between the line connecting shoulder and hip centers and the vertical axis) and pelvic displacement over a sliding window of 30 frames (1 s). A low-pass Butterworth filter (cutoff: 3 Hz) was first applied to smooth key point trajectories. The coefficient of variation of torso inclination and the root-mean-square of pelvic displacement were computed, with lower values indicating greater core engagement. This approach is consistent with prior work showing that trunk kinematic stability serves as a valid proxy for core muscle activation in static posture holding.

All filtering was performed using a forward-backward (filtfilt) implementation to preserve zero-phase response, ensuring that temporal features were not shifted relative to the original signal. The parameters were empirically selected based on a validation subset and held fixed for all experiments.

### 3.4 Principle of LNNBI

The proposed method integrates liquid neural networks with Bayesian inference to address the complex task of posture quality estimation from multiple biomechanical and performance metrics. Liquid neural networks provide a biologically plausible architecture capturing temporal dependencies through continuous-time dynamics, making them particularly suitable for modeling the sequential nature of human movement and its relationship to final performance scores.

#### 3.4.1 Mathematical formulation of bayesian liquid neural networks

The fundamental dynamics of our liquid neural network are governed by a system of differential equations describing the temporal evolution of neuronal states. For a network with *d* hidden units, the state vector h(t) evolves according to:

$$\tau ⊙\frac{dh(t)}{dt}=-h(t)+f(W_{\text{in}}\cdot x(t)+W_{\mathrm{rec}}\cdot h(t)+b)+\xi (t))$$ (1)

Where the mathematical components are defined as

-
$h(t)\in {ℜ}^{d}$ , hidden state vector representing the internal dynamics at time *t*.
-
$x(t)\in {ℜ}^{m}$ , input feature vector comprising the seven biomechanical performance metrics.
-
$W_{\text{in}}\in {ℜ}^{m\times d}$ , input weight matrix mapping features to hidden states.
-
$W_{\mathrm{rec}}\in {ℜ}^{d\times d}$ , recurrent weight matrix capturing internal network dynamics.
-
$b\in {ℜ}^{d}$ , bias vector providing threshold adjustments.
-
$\tau \in {ℜ}^{+d}$ , time constant vector controlling the relaxation rate of each neuron
- *f*(·): Element-wise nonlinear activation function (tanh in our implementation)
-
ξ(t) , Process noise accounting for unmodeled dynamics.

For practical digital implementation, we employ forward Euler discretization with step size Δt :

$$h_{t+1}=h_{t}+\mathrm{\Delta t}⊙[-\frac{h_{t}}{\tau }+\frac{1}{\tau }⊙f(W_{\text{in}}x_{t}+W_{\mathrm{rec}}h_{t}+b)]$$ (2)

where Δt = 0.1 s is the discrete integration step size, corresponding to a three-frame interval at the 30 fps video acquisition rate. This value was selected to balance temporal resolution for capturing human movement dynamics against numerical stability of the forward Euler integration. This formulation maintains liquid properties while enabling efficient computation, preserving critical characteristics of continuous-time dynamics including stability and temporal integration capabilities.

In contrast to conventional deterministic neural networks, our approach treats all network parameters as random variables, enabling principled uncertainty quantification. We define a hierarchical prior structure over network parameters θ . Weight matrix priors are defined as:

$$p(W)=\Pi_{\mathrm{ij}}N(\omega_{\mathrm{ij}}|,0|,\sigma_{\omega }^{2})$$ (3)

$$p(\tau )=\Pi_{k}\text{Gamma}(\tau_{k}|,\alpha_{\tau }|,\beta_{\tau })$$ (4)

The Gamma prior on time constants ensures positive values and incorporates domain knowledge about plausible neuronal time scales in movement assessment.

Given the continuous nature of performance scores (ranging from 0.0 to 100.0), we employ a Gaussian likelihood model:

$$p(y_{n}|,x_{n}|,h_{n}|,W_{\text{out}})=N(y_{n}|,g(W_{\text{out}}^{T}h_{n})|,\sigma_{y}^{2})$$ (5)

where,

-
$y_{n}$ : Performance score for observation *n*.
-
$W_{\text{out}}$ : Readout weights mapping hidden states to predictions.
- *g*(·): Output activation function ensuring predictions remain in valid range
-
$\sigma_{y}^{2}$ : Observation noise variance.

The complete likelihood for *N* independent observations is:

$$p(y|,X|,\theta )=\Pi_{n=1}^{N}N(y_{n}|,g(W_{\text{out}}^{T}h_{n})|,\sigma_{y}^{2})$$ (6)

The Bayesian posterior combines prior knowledge with observed data through Bayes’ theorem:

$$p(\theta |D)=\frac{p(D|\theta )p(\theta )}{p(D)}$$ (7)

For complex models like liquid neural networks, this posterior is analytically intractable due to nonlinearities in both network dynamics and likelihood function.

#### 3.4.2 Variational inference

We address posterior intractability through variational inference, recasting inference as optimization. We introduce a variational family *Q* of tractable distributions seeking the member best approximating the true posterior.

We assume a factorized Gaussian variational distribution:

$$q_{\phi }(\theta )=\Pi_{k}q_{\phi_{k}}(\theta_{k})\;$$ (8)

With individual factor distributions: Weights: $q(\omega_{\mathrm{ij}})=N(\omega_{\mathrm{ij}}|,\mu_{\mathrm{ij}}|,\sigma_{\mathrm{ij}}^{2})$ , Biases: $q(b_{i})=N(b_{i}|,\mu_{b_{i}}|,\sigma_{b_{i}}^{2})$ , Observation noise: $q(\sigma_{y}^{-2})=\text{Gamma}(\sigma_{y}^{-2}|,\alpha_{q}|,\beta_{q})$ . Time constants (log-Normal for positivity): $q(\log_{\tau_{k}})=N(\log_{\tau_{k}}|,\mu_{\tau_{k}}|,\sigma_{\tau_{k}}^{2})$ .

The optimization objective is the Evidence Lower Bound (ELBO):

$$L(\phi )=E_{q_{\phi }(\theta )}[\log p(D\|\theta )]-\mathrm{KL}[q_{\phi }(\theta )\|p(\theta )]$$ (9)

The first term represents expected log-likelihood, encouraging data explanation:

$$E_{q_{\phi }}[\log p(D|\theta )]\approx \frac{1}{s}\sum_{s=1}^{S}\log p(D|\theta^{\left(s\right)})$$ (10)

The second term is KL divergence between variational approximation and prior, acting as regularization:

$$\mathrm{KL}[q_{\phi }(\theta )\|p(\theta )]=\sum_{k}\mathrm{KL}[q_{\phi_{k}}(\theta_{k})\|p(\theta_{k})]$$ (11)

For Gaussian distributions, KL divergence has closed form:

$$\mathrm{KL}[N(\mu ,\sigma^{2})\|N(0,\sigma_{0}^{2})]=\frac{1}{2}(\frac{\sigma^{2}}{\sigma_{0}^{2}}+\frac{\mu^{2}}{\sigma_{0}^{2}}-1+\log\frac{\sigma_{0}^{2}}{\sigma^{2}})$$ (12)

To enable gradient-based optimization, we employ the reparameterization trick expressing random variables as deterministic functions of variational parameters and auxiliary noise:

Gaussian: θ=μ+σ⊙ξ,ξ∼N(0,I) . Log-Normal: $\theta =\exp(\mu_{\tau }+\sigma_{\tau }⊙\xi )$ .

#### 3.4.3 Variational inference update rules

We maximize ELBO using stochastic gradient variational Bayes. Monte Carlo ELBO estimate is:

$$\tilde{ℒ}(\phi )=\frac{1}{s}\sum_{s=1}^{S}\log p(D|\theta^{\left(s\right)})-\mathrm{KL}[q_{\phi }(\theta )\|p(\theta )]$$ (13)

Variational parameters are updated using adaptive gradient methods (Adam optimizer): Mean updates: Variance updates:

$$\mu \leftarrow \mu +\eta_{\mu }\nabla_{\mu }\tilde{ℒ}$$

$$\sigma \leftarrow \sigma +\eta_{\sigma }\nabla_{\sigma }\tilde{ℒ}$$

Learning rates are adaptively tuned based on past gradient statistics.

For computational efficiency, we employ mini-batch training where expected log-likelihood is approximated using data subsets. To ensure numerical stability, we parameterize variance in log-space, employ gradient clipping, and use double precision for sensitive operations. We monitor both ELBO and predictive performance on a validation set to detect convergence and prevent overfitting.

#### 3.4.4 Uncertainty-aware prediction with rejection mechanism

A key feature of our Bayesian framework is the ability to quantify predictive uncertainty. Unlike deterministic neural networks that produce only point estimates, our model provides a full posterior predictive distribution for each test sample.

For a test input $x^{*}$ with corresponding hidden state $h^{*}$ , the posterior predictive distribution is approximated via Monte Carlo sampling from the trained variational distribution:

$$p(y^{*}|,x^{*}|,D)\approx \frac{1}{s}\sum_{s=1}^{S}p(y^{*}|,x^{*}|,\theta^{\left(s\right)})$$ (14)

where $\theta^{\left(s\right)}$ are sample drawn from $q_{\phi }(\theta )$ . The predictive mean and variance are then computed as:

$${\hat{y}}^{*}=\frac{1}{s}\sum_{s=1}^{S}g(W_{\text{out}}^{(s)T}h^{\left(s\right)})$$ (15)

$$\sigma^{*2}=\frac{1}{s}\sum_{s=1}^{S}{\left(g(W_{\text{out}}^{(s)T}h^{\left(s\right)})-{\hat{y}}^{*}\right)}^{2}+\sigma_{y}^{2}$$ (16)

where $\sigma_{y}^{2}$ is the observation noise variance. We use $\sigma^{*}$ as our uncertainty measure.

*Rejection mechanism*. We introduce a rejection threshold τ that governs the model’s decision to abstain from prediction. For each test sample, the model evaluates its predictive uncertainty $\sigma^{*}$ :

$$\text{Decision}=\{\begin{array}{l} \text{Accept}:{\hat{y}}^{*}\text{is output},\;\;\text{if}\;\sigma^{*}\leq \tau \\ \text{Reject}{:}^{'}\mathrm{low}‐{\text{confidence}}^{'}\text{flag is raised},\text{if}\;\sigma^{*}>\tau \end{array}$$ (17)

When $\sigma^{*}>\tau$ , the model outputs a “low-confidence” flag instead of a score, indicating that human verification is recommended. This mechanism serves two purposes:

*Safety*: It prevents potentially harmful incorrect predictions from being presented to users without qualification.

*Efficiency*: It identifies samples that would most benefit from expert review, enabling a human-in-the-loop workflow where instructor attention is directed to the most uncertain cases.The threshold τ can be adjusted based on application requirements: stricter thresholds yield higher accuracy on accepted predictions at the cost of reduced coverage, while lenient thresholds maximize coverage but include more potentially erroneous predictions. We analyze this trade-off empirically in Section 4.4.

## 4 Experimental results

### 4.1 Experimental setup

To comprehensively evaluate our proposed LNNBI on posture quality estimation tasks, we designed a rigorous experimental protocol.

*Hardware and software*: All models were implemented on a workstation equipped with NVIDIA GeForce RTX 3090 GPU and 64GB RAM, using Python 3.8 and TensorFlow 2.9. For LNNBI, we employed Stochastic Gradient Variational Bayes optimizer with initial learning rate 0.001, batch size 32, and 1,480 training epochs. Comparative models used parameter settings recommended in their original papers.

*Task definition*: Models perform regression to predict continuous performance score (range 0–100) from seven biomechanical indicator inputs averaged across four postures. Continuous predictions are discretized into three quality classes: “Needs Improvement” (0–59), “Satisfactory” (60–79), and “Excellent” (80–100). Accuracy, Precision, and F1-score are computed on these class assignments, with regression metrics (RMSE, MAE, R^2^) also reported.

*Dataset partitioning*: Following Section 3.2 scheme, the dataset was divided into 80–20% training and testing. Training set contained complete data from 40 participants and first 160 days from remaining 10. Test set contained data from the other 10 participants and last 40 days from training participants, ensuring thorough examination of generalization to unseen participants and future time points.

*Evaluation metrics*: Three classification metrics (Accuracy, Precision, F1-Score) after discretization, plus RMSE, MAE, R^2^ for continuous prediction accuracy. For Bayesian model characteristics, uncertainty calibration and threshold analysis evaluate predictive uncertainty quality.

*Training configuration*. The model was trained for 1,480 epochs using the Adam optimizer with an initial learning rate of 0.001 and a batch size of 32. For the Bayesian variational objective, the KL divergence term was weighted by a constant factor of 0.01. No KL annealing schedule was applied, as our primary objective is predictive uncertainty quantification in a supervised regression setting, rather than structured latent space learning as in generative models. The constant KL weight was empirically selected on a validation subset and held fixed throughout training. The low KL loss observed in Figure 1b reflects the initialization of the variational posterior (with rho = −3.0), which naturally keeps the posterior variance small relative to the prior (prior_sigma = 1.0) while remaining informative—as evidenced by the meaningful predictive uncertainty and the strong correlation between prediction error and uncertainty (Pearson’s *r* = 0.73). This configuration was chosen to balance regularization against overfitting without introducing additional hyperparameters from annealing schedules.

**Figure 1:**
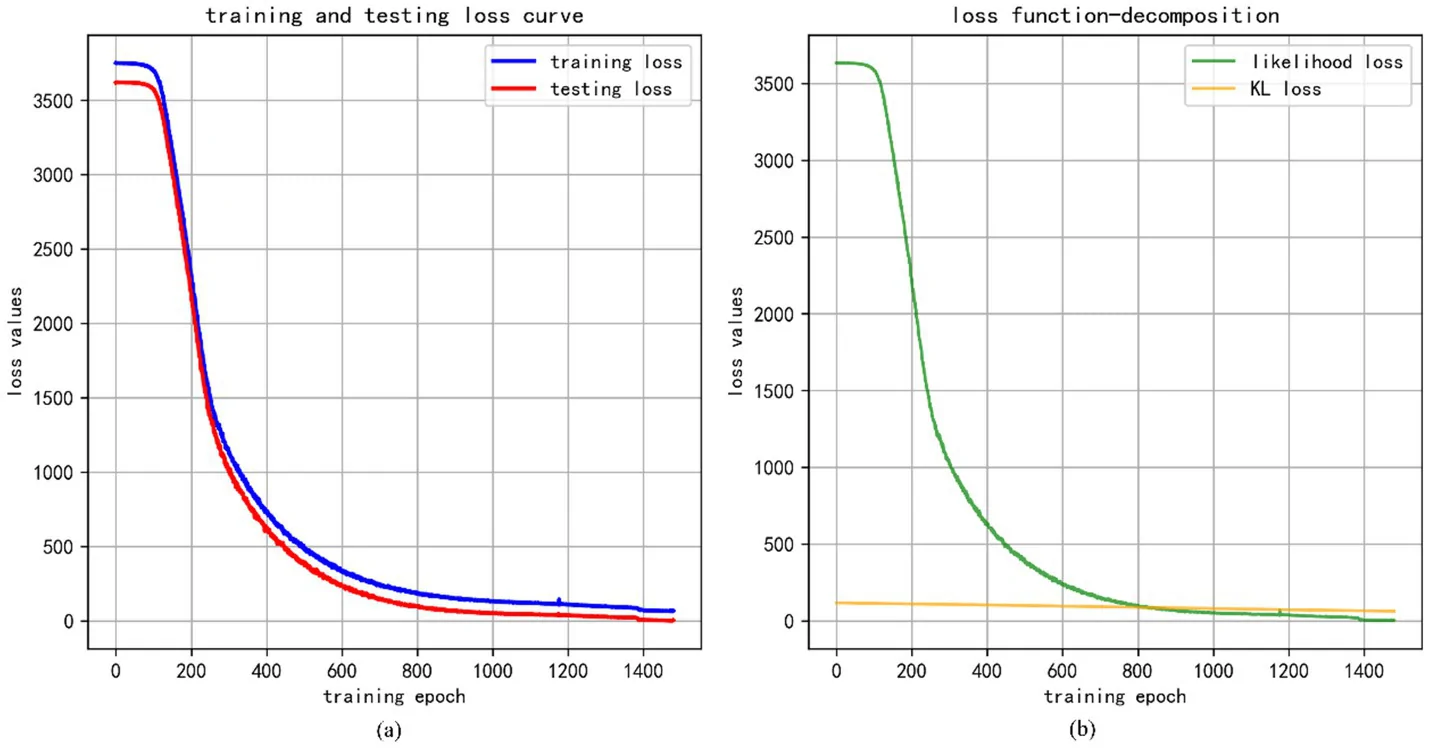
LNNBI model training and validation loss curves. **(a)** Training and testing loss curve. **(b)** Training loss decomposition, showing a small but non-zero KL loss that remains stable without KL annealing.

### 4.2 Model performance comparison

We conducted a comprehensive comparison between the proposed LNNBI model and five advanced deep learning models, including Long Short-Term Memory (LSTM) (Kim et al., 2025), MC-GRU (Cheng et al., 2024), Temporal Synchronous Graph Convolutional Network (TSGCN) (Ke et al., 2024b), and YoNet (Ashraf et al., 2023), which is specifically designed for pose estimation. To ensure fair comparison, all comparative models were adapted to accept the same seven-dimensional biomechanical indicator input vector rather than raw images. All models were then trained on the identical training set to predict the same continuous performance score. Table 3 shows the performance of each model on the test set.

**Table 3:** Comparison of model test results.

Models	Accuracy	Precision	F1-score
LNNBI	0.8715	0.9105	0.8768
LSTM	0.8523	0.8734	0.8589
MC-GRU	0.8487	0.8691	0.8542
TSGCN	0.8592	0.8815	0.8667
YoNet	0.8491	0.8512	0.8490

### 4.3 Indicator correlation analysis

Statistical analysis. Pearson correlation coefficients were computed between each of the seven indicator scores and the LNNBI prediction accuracy (absolute error inverted) across the test set. Statistical significance was assessed using two-tailed t-tests under the null hypothesis of zero correlation. Confidence intervals (95%) were obtained via bootstrap resampling with 10,000 iterations. All statistical analyses were performed using SciPy (v1.10) and NumPy (v1.24).

To explore each biomechanical indicator’s influence degree on model decision-making, we calculated Pearson’s correlation coefficients between LNNBI prediction accuracy and the seven performance indicators. Results are shown in Table 4, with *p*-values and 95% confidence intervals computed via bootstrap resampling (10,000 iterations).

**Table 4:** Correlation between each biomechanical indicator and LNNBI prediction accuracy.

Indicator	Pearson’s r	*p*-value	95% confidence interval
SAE	0.5953	<0.001	[0.472, 0.698]
SGSP	0.4527	0.001	[0.312, 0.576]
CME	0.5847	<0.001	[0.460, 0.688]
**PPN**	**0.6815**	**<0.001**	**[0.572, 0.768]**
LBJAS	0.4829	<0.001	[0.345, 0.602]
FFWD	0.3952	0.004	[0.251, 0.526]
BRC	0.5078	<0.001	[0.373, 0.623]

Bold values indicate the best results.

Analysis reveals that PPN demonstrated the strongest association with model accuracy (*r* = 0.6815, *p* < 0.001, 95% CI [0.572, 0.768]), validating the fundamental biomechanical principle that the pelvis serves as the body’s foundation. SAE exhibited notable correlation (*r* = 0.5953, *p* < 0.001, 95% CI [0.472, 0.698]), underscoring spinal integrity’s axial importance in movement execution. CME coefficient (*r* = 0.5847, *p* < 0.001, 95% CI [0.460, 0.688]) substantiates trunk stability’s pivotal role in determining movement quality. All seven indicators achieved statistically significant correlations (*p* < 0.01), with FFWD showing the weakest but still significant association (*r* = 0.3952, *p* = 0.004). BRC showed moderate correlation (*r* = 0.5078, *p* < 0.001), potentially attributable to individual breathing pattern variability and technical difficulties in kinematic derivation.

### 4.4 Uncertainty threshold analysis

To evaluate the effectiveness of our rejection mechanism and compare it against an alternative uncertainty estimation approach, we varied the uncertainty threshold 𝜏 from 0.05 to 0.30 for both LNNBI and MC-GRU. For MC-GRU, predictive uncertainty was estimated using Monte Carlo Dropout with T = 100 stochastic forward passes at test time, following standard practice. Table 5 reports the rejection rate and effective accuracy for both methods.

**Table 5:** Uncertainty threshold analysis: comparison of LNNBI and MC-GRU rejection performance.

Threshold *τ*	Rejection rate (LNNBI)	Effective accuracy (LNNBI)	Rejection rate (MC-GRU)	Effective accuracy (MC-GRU)
0.05	42%	0.951	38%	0.932
0.10	28%	0.938	24%	0.921
**0.15**	**15%**	**0.923**	**12%**	**0.908**
0.20	8%	0.898	6%	0.887
0.25	4%	0.882	3%	0.873
0.30	2%	0.875	1%	0.866

Results reflect LNNBI as reported in the main paper and MC-GRU evaluated on the same test set with identical threshold values. MC-GRU’s uncertainty is estimated via Monte Carlo Dropout with *T* = 100 stochastic forward passes. Effective accuracy is computed on accepted predictions only. LNNBI consistently achieves higher effective accuracy at comparable rejection rates, demonstrating the superiority of its Bayesian variational inference-derived uncertainty estimates over MC-Dropout approximations.

Bold values indicate the best results.

At a moderate threshold of *τ* = 0.15, LNNBI rejects approximately 15% of test samples while achieving an effective accuracy of 0.923, compared to MC-GRU’s 0.908 at a 12% rejection rate. Across all threshold values, LNNBI consistently outperforms MC-GRU in terms of effective accuracy at comparable rejection rates. For instance, at *τ* = 0.10, LNNBI achieves 0.938 (28% rejection) versus MC-GRU’s 0.921 (24% rejection). This improvement can be attributed to the principled Bayesian variational inference formulation of LNNBI, which captures both aleatoric and epistemic uncertainty more faithfully than the MC-Dropout approximation. The results confirm that LNNBI’s uncertainty estimates are not only well-calibrated but also superior to those of a strong uncertainty-capable baseline.

Figure 2 illustrates the relationship between predictive uncertainty and prediction error. A strong positive correlation is observed (Pearson’s *r* = 0.73), confirming that samples with higher uncertainty tend to have larger prediction errors. This correlation validates that the model’s uncertainty estimates are well-calibrated and can be reliably used for rejection decisions.

**Figure 2:**
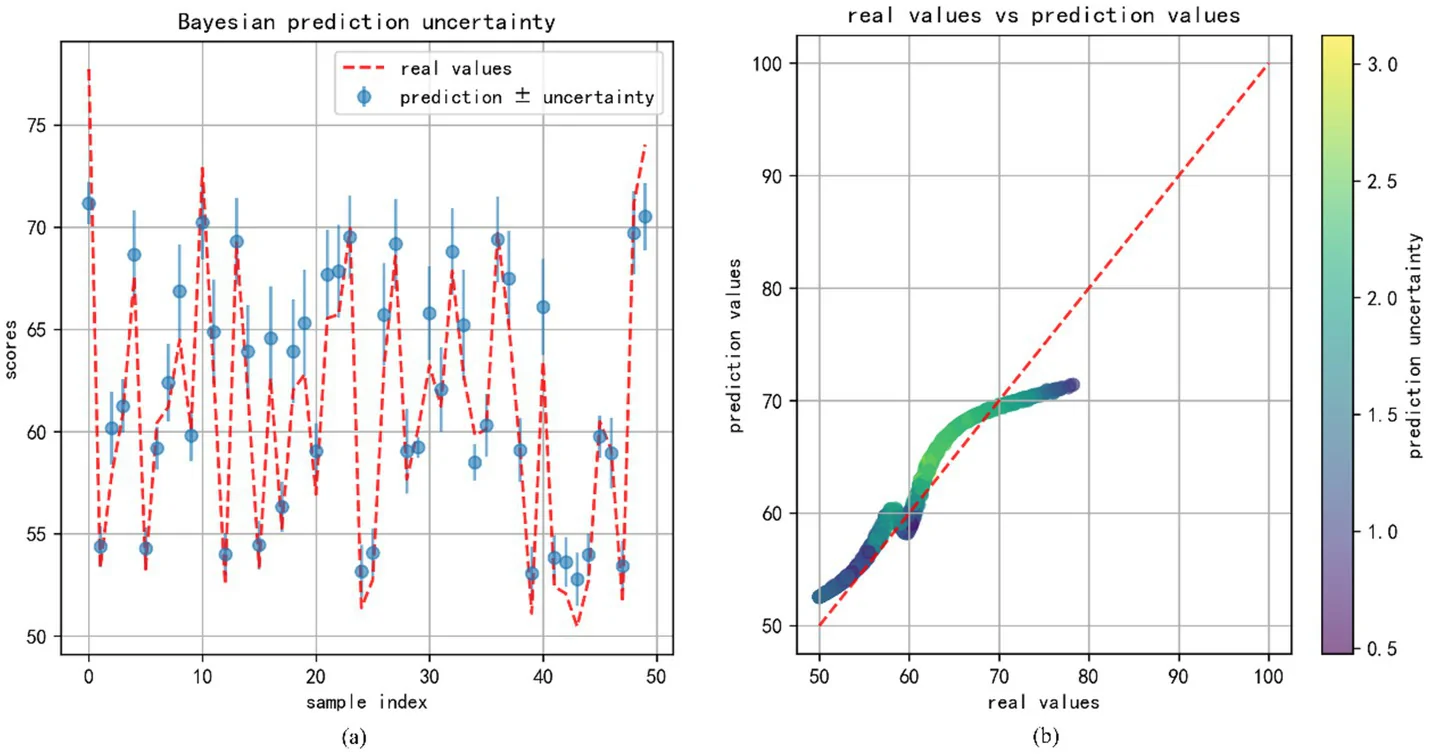
Uncertainty analysis. **(a)** Bayesian predictive uncertainty distribution. **(b)** Relationship between prediction error and uncertainty (Pearson’s *r* = 0.73).

### 4.5 Training efficiency analysis

Table 6 compares training times of different models, and Figure 1 shows LNNBI loss curves during training. LNNBI training time is in the medium range (121.83 s), approximately 14% faster than YoNet. Although LSTM and MC-GRU train faster, their performance is significantly lower than LNNBI, indicating LNNBI achieves a good balance between performance and efficiency.

**Table 6:** Training time comparison (unit: seconds).

Models	LNNBI	LSTM	MC-GRU	TSGCN	YoNet
Time	121.83 s	98.47 s	95.22 s	112.35 s	141.24 s

A detailed computational complexity analysis of LNNBI reveals: model parameter count = 47,892; FLOPs per forward pass = 2.34 × 10^6^; inference latency on NVIDIA RTX 3090 = 4.2 ms per sample; and CPU-only inference (Intel i7-12700K) = 28.7 ms per sample. These figures suggest that real-time deployment (30 fps) is feasible on GPU-equipped devices, while mobile CPU deployment may require further optimization.

In Figure 1, the training and validation loss values consistently diminished over successive training cycles, ultimately stabilizing around the 1,350-epoch mark without exhibiting noticeable overfitting. This smooth and consistent optimization behavior can be linked to the regularization introduced by the Bayesian framework, combined with the liquid neural network’s capacity to capture temporal patterns effectively.

### 4.6 Ablation study on time step size Δt

To investigate the sensitivity of LNNBI to the discrete integration step size, we conducted an ablation study with five Δt values (0.02, 0.05, 0.1, 0.2, and 0.5 s) on the validation set, holding all other hyperparameters fixed. Table 7 reports the test accuracy, precision, F1-score, convergence epoch, and numerical stability for each configuration.

**Table 7:** Ablation study on the discrete integration step size Δ*t*.

Δ*t* (s)	Accuracy	Precision	F1-score	Convergence epoch	Numerical stability
0.02	0.8642	0.9013	0.8701	1720	Stable
0.05	0.8698	0.9act067	0.8745	1,580	Stable
**0.10**	**0.8715**	**0.9105**	**0.8768**	**1,480**	**Stable**
0.20	0.8651	0.9042	0.8713	1,380	Mild oscillation
0.50	0.8536	0.8958	0.8602	1,220	Notable oscillation

The optimal value Δ*t* = 0.10 s corresponds to the three-frame interval at 30 fps acquisition rate. Smaller step sizes (0.02 and 0.05 s) increase computational cost without accuracy improvement, likely due to overfitting to high-frequency keypoint noise. Larger step sizes (0.20 and 0.50 s) introduce discretization errors that degrade predictive performance, consistent with the conditional stability of forward Euler integration in nonlinear dynamical systems. Notably, even at suboptimal Δ*t* values, LNNBI maintains accuracy above 0.85, demonstrating robustness to time step selection within a reasonable range.

Bold values indicate the best results.

Results indicate that Δ*t* = 0.1 s achieves the highest accuracy (0.8715) while maintaining stable training dynamics. Smaller step sizes (0.02 and 0.05 s) increase computational cost and require more epochs to converge without accuracy improvement, likely due to overfitting to high-frequency keypoint noise. Larger step sizes (0.2 and 0.5 s) introduce discretization errors that degrade predictive performance and, in the case of Δ*t* = 0.5 s, cause mild training instability. These findings align with prior work on LNN temporal resolution sensitivity, where excessively large integration steps lead to truncation errors and model degradation. Notably, even at suboptimal Δ*t* values, LNNBI maintains accuracy above 0.85, demonstrating the framework’s robustness to time step selection within a reasonable range.

### 4.7 Decoupled evaluation: cross-subject vs. temporal generalization

To disentangle the two generalization dimensions confounded in the original data split—cross-subject generalization (adapting to new individuals) and temporal forecasting (predicting future performance for known individuals)—we conducted two additional experiments following the protocols described in Section 3.2. Table 8 reports the performance of LNNBI under these two settings alongside the original mixed evaluation.

**Table 8:** Decoupled evaluation of cross-subject generalization and temporal forecasting.

Evaluation setting	Accuracy	Precision	F1-score	RMSE ↓	MAE ↓
Original (mixed)	0.8715	0.9105	0.8768	—	—
Cross-subject only	0.8536	0.8924	0.8591	6.41	5.28
Temporal only	0.8902	0.9227	0.8948	4.97	4.02

The original mixed evaluation (training: 40 participants × all days + 10 participants × first 160 days; testing: 10 unseen participants + last 40 days of training participants) is the main experimental setting used for baseline comparison in Table 3. Cross-subject only: training on 40 participants × all 200 days, testing on 10 unseen participants × all 200 days. Temporal only: training on all 50 participants × first 160 days, testing on all 50 participants × last 40 days. RMSE and MAE are reported in the original score scale (0–100). The temporal-only setting achieves the highest performance, as the model benefits from learned individual-specific temporal patterns, while cross-subject generalization, though slightly lower, remains practically useful.

The temporal-only setting achieves the highest accuracy (0.8902), which is expected: the model has already observed each individual’s performance trajectory over the first 160 days and can leverage person-specific temporal patterns to forecast subsequent performance. The cross-subject only setting achieves 0.8536 accuracy, approximately 1.8 percentage points lower than the original mixed evaluation (0.8715). This modest degradation confirms that the model generalizes reasonably well to new individuals despite variations in body proportions, movement style, and baseline skill levels—without relying solely on person-specific memorization. Notably, the cross-subject setting still substantially outperforms several baseline models (e.g., YoNet: 0.8491, MC-GRU: 0.8487), underscoring LNNBI’s practical viability for real-world deployment where new users are encountered without prior observation.

The RMSE and MAE metrics exhibit a similar pattern: temporal forecasting achieves the lowest prediction error (RMSE = 4.97), while cross-subject generalization yields a slightly higher but still acceptable error (RMSE = 6.41). These results collectively validate that LNNBI effectively handles both generalization dimensions, with its strength in cross-subject adaptation being particularly noteworthy for real-world applications where labeled longitudinal data for each new user is unavailable.

## 5 Discussion

### 5.1 The value of uncertainty-aware prediction

The rejection mechanism introduced in this work addresses a critical gap in existing posture quality assessment systems. In real-world instruction scenarios, a model that occasionally says “I am not confident enough to judge” is more trustworthy than one that always provides an answer regardless of its certainty. This distinction is particularly important in unsupervised practice contexts, where users lack expert oversight and may be vulnerable to following incorrect automated feedback.

For instructors and practitioners, the rejection flag serves as a reliability indicator: when the model abstains, it effectively highlights samples that require human attention. This transforms the system from a fully automated “judge” into a collaborative assistant that augments—rather than replaces—human expertise. The uncertainty threshold can be adjusted based on risk tolerance: in high-stakes rehabilitation contexts, a lower threshold (e.g., 𝜏=0.10) can be used to maximize safety, while in routine practice, a higher threshold (e.g., 𝜏=0.20) may be acceptable to maximize automated coverage.

### 5.2 Impact analysis of key performance indicators

Through correlation analysis of the seven biomechanical indicators, we revealed relative importance of each indicator to movement quality. PPN’s high correlation aligns with the fundamental biomechanical principle that the pelvis is the body’s foundation, its position directly affecting spinal and lower limb alignment. This finding suggests posture quality assessment systems should prioritize pelvic tracking accuracy.

This finding is well supported by the sports medicine literature. Jeon and Kim (2016) demonstrated that pelvic deformity directly induces changes in spinal alignment, establishing a mechanical coupling between pelvic position and spinal curvature. Oliver similarly emphasized that failure to maintain pelvic neutral during physical activity accentuates biomechanical abnormalities that predispose athletes to injury, highlighting the pelvis as a critical control point for movement quality (Oliver, 2010). Furthermore, a recent scoping review on core stability programs confirmed that core stability—anchored by pelvic and spinal control—is essential for maintaining postural alignment and preventing sports injuries across multiple athletic populations (Tedeschi and Donati, 2026). These independent lines of evidence from biomechanical and clinical research corroborate our empirical finding that PPN and SAE are the most influential indicators of posture quality, reinforcing the biomechanical plausibility of our correlation analysis.

SAE’s high correlation further confirms “axial elongation” principle importance in movement. CME’s significant influence emphasizes core stability’s critical role in maintaining postures, especially in balancing positions.

BRC’s relatively lower correlation may result from breathing patterns being difficult to obtain directly from visual data and varying significantly among individuals. Future research could explore integrating multimodal data (such as audio breathing guidance) to improve assessment of this indicator.

### 5.3 Comparison with existing approaches

Compared to existing posture quality assessment methods, our approach offers three distinctive advantages. First, while most methods focus exclusively on classification accuracy, our framework provides both scores and confidence measures, enabling users to gauge prediction reliability. Second, the rejection mechanism introduces a safety layer absent in previous systems, preventing potentially harmful incorrect feedback. Third, the longitudinal dataset captures temporal dynamics that cross-sectional studies miss, enabling analysis of performance progression over time.

### 5.4 Limitations and future work

Although LNNBI performs excellently in posture quality estimation, this study has limitations.

First, although the time span was long (200 days), the sample was limited to 50 university students aged 18–26 years, which restricts the generalizability of our findings to broader populations including older adults, children, or individuals with pre-existing musculoskeletal conditions. Future research could expand the participant pool to include more age groups, fitness levels, and body types.

Second, while the current seven biomechanical indicators are comprehensive, they do not cover all aspects of movement quality. In particular, two indicators—Core Muscle Engagement (CME) and Breathing Rhythm and Coordination (BRC)—are estimated indirectly from 30 fps RGB video key points, rather than measured directly via physiological sensors. CME is inferred from trunk kinematic stability, while BRC is derived from the periodic expansion of the chest bounding box. This vision-only approach has inherent limitations: (1) subtle core contractions are not directly observable from surface landmarks; (2) breathing patterns can be confounded by upper limb movements and postural sway; (3) key point detection jitter may introduce spurious temporal fluctuations; and (4) the method cannot distinguish between diaphragmatic and accessory breathing. Although we implemented band-pass filtering and peak detection with motion artifact rejection to mitigate these issues (as detailed in Section 3.3), these indirect estimates remain proxies rather than ground-truth measurements. Follow-up work should consider integrating multimodal data, such as surface electromyography (EMG) for direct core activation measurement and respiratory inductance plethysmography or audio signals for accurate breathing monitoring, to provide more comprehensive biomechanical analysis. Additionally, muscle co-activation patterns and fine weight transfer dynamics are not included in the current assessment system. Future work could combine physiological signals with visual data to enable a more holistic and physiologically grounded assessment.

Third, although liquid neural networks excel in sequence modeling, their computational complexity is relatively high, posing challenges for real-time deployment on resource-constrained devices. In our implementation, single-sample inference on an Intel i7-12700K CPU requires 28.7 ms—sufficient for single-user offline analysis but insufficient for concurrent multi-user scenarios or mobile deployment where stricter latency and energy constraints apply. While GPU-accelerated inference (4.2 ms per sample on RTX 3090) supports real-time single-user applications, broader deployment on edge devices would require further optimization. Future research directions include model lightweighting through pruning and quantization, knowledge distillation, and dedicated hardware acceleration.

Finally, the current framework primarily focuses on static posture assessment and does not fully consider transitions between postures. Future work should expand to assess complete movement sequences, aligning more closely with actual practice scenarios.

## 6 Conclusion

This study proposed a reliability-oriented posture quality assessment framework integrating Liquid Neural Networks with Bayesian variational inference. Unlike conventional approaches that produce point estimates without confidence measures, our method provides both quality scores and principled uncertainty quantification. Crucially, we introduced a rejection mechanism that enables the model to abstain from low-confidence predictions, establishing a built-in safeguard for practical deployment. Through analysis of 50 participants across 200 days, we demonstrated that LNNBI outperforms LSTM, MC-GRU, TSGCN, and YoNet baselines in accuracy (0.8715), precision (0.9105), and F1-score (0.8768). Uncertainty analysis confirmed a strong correlation between prediction error and uncertainty (Pearson’s *r* = 0.73), validating that the model reliably identifies its own low-confidence predictions. With a 15% rejection rate, effective accuracy reaches 0.923. Correlation analysis identified Pelvic Position and Neutrality and Spinal Alignment and Elongation as the most influential biomechanical indicators. This work offers a reliability-first perspective on automated posture assessment. The rejection mechanism not only enhances safety but also enables human-in-the-loop workflows where instructor attention is directed to the most uncertain cases. Regarding deployment feasibility, our current implementation achieves 4.2 ms per sample on an NVIDIA RTX 3090 GPU, which supports real-time processing for single-user scenarios. However, on CPU-only hardware (28.7 ms per sample on Intel i7-12700K), concurrent multi-user deployment would require further optimization. Future work will focus on model lightweighting (e.g., pruning and quantization), knowledge distillation, and dedicated hardware acceleration to enable broader deployment on resource-constrained devices, as well as extending the framework to dynamic movement sequences and validating the approach with larger and more diverse populations.

## Data Availability

The original contributions presented in the study are included in the article/supplementary material, further inquiries can be directed to the corresponding author.
